# Cerebellar Atrophy and Language Processing in Chronic Left-Hemisphere Stroke

**DOI:** 10.1162/nol_a_00120

**Published:** 2024-08-15

**Authors:** Roger D. Newman-Norlund, Makayla Gibson, Lisa Johnson, Alex Teghipco, Chris Rorden, Leonardo Bonilha, Julius Fridriksson

**Affiliations:** Department of Psychology, University of South Carolina, Columbia, SC, USA; Department of Communication Sciences, University of South Carolina, Columbia, SC, USA; School of Medicine, University of South Carolina, Columbia, SC, USA

**Keywords:** brain, cerebellum, chronic stroke, language

## Abstract

Chronic stroke results in significant downstream changes at connected cortical sites. However, less is known about the impact of cortical stroke on cerebellar structure. Here, we examined the relationship between chronic stroke, cerebellar volume, cerebellar symmetry, language impairment, and treatment trajectories in a large cohort (*N* = 249) of chronic left hemisphere (LH) stroke patients with aphasia, using a healthy aging cohort (*N* = 244) as control data. Cerebellar gray matter volume was significantly reduced in chronic LH stroke relative to healthy control brains. Within the chronic LH stroke group, we observed a robust relationship between cerebellar volume, lesion size, and days post-stroke. Notably, the extent of cerebellar atrophy in chronic LH patients, particularly in the contralesional (right) cerebellar gray matter, explained significant variability in post-stroke aphasia severity, as measured by the Western Aphasia Battery—Revised, above and beyond traditional considerations such as cortical lesion size, days post-stroke, and demographic measures (age, race, sex). In a subset of participants that took part in language treatment studies, greater cerebellar gray matter volume was associated with greater treatment gains. These data support the importance of considering both cerebellar volume and symmetry in models of post-stroke aphasia severity and recovery.

## INTRODUCTION

A stroke occurs every 40 seconds in the United States and the total economic burden of stroke is estimated to reach over 240 billion USD per year by the year 2030 (American Heart Association, 2023; Ovbiagele et al., 2013). Aphasia, which most commonly results from insult to the left middle cerebral artery, can have a profound effect on the quality of life (Spaccavento et al., 2014) and mental health (Gainotti, 1997) of those affected as well as their caregivers (Michallet et al., 2003). While lesion size and location are typically reliable predictors of aphasia severity and behavioral outcomes (Busby et al., 2023; Kristinsson et al., 2022; Thye & Mirman, 2018), researchers have also identified important functional and structural changes at cortical sites connected to, but distal from, the original lesion site (Baldassarre et al., 2016; Kalinosky et al., 2017; Tang et al., 2016; Wilmskoetter et al., 2019, 2021). However, the relationship between structural integrity of the cerebellum, aphasia severity and the potential for recovery of language abilities in chronic left hemisphere (LH) stroke has not been systematically evaluated.

The lack of data relating cerebellar integrity to stroke is striking given what is known about the interconnectedness of the cerebellum and putative language areas (Justus, 2004; Leiner et al., 1993; Silveri et al., 1994; Zettin et al., 1997). Based on these and other studies, cerebellar lesions are hypothesized to impact human language via polysynaptic pathways related to, but not overlapping with, core linguistic systems (Fiez, 2016; Moberget & Ivry, 2016). Complementary evidence for cerebellar involvement in language processing comes from various functional imaging studies. For example, Petersen and colleagues (1989) used positron emission tomography to identify right cerebellar activation during verb generation, which, at the time, was thought to reflect retrieval and preparation of linguistic motor responses. Data from functional magnetic resonance imaging (fMRI) studies provide further evidence that the cerebellum is critically involved in language (Booth et al., 2007; Desmond & Fiez, 1998; Xiang et al., 2003), with a recent meta-analysis concluding that specific regions of the cerebellum, including Crus I, Crus II, as well as Lobules VI and VIIA, are particularly integral to language function (Mariën & Manto, 2015).

In the current study, we leveraged a large and relatively homogeneous population of chronic LH stroke survivors with aphasia (*N* = 249), along with control data from our local Aging Brain Cohort Repository (Newman-Norlund et al., 2021; *N* = 244), to examine the relationship between cerebellar changes in individuals with aphasia as a result of chronic LH stroke (Table 1). Cerebellar structural integrity was quantified as (1) gray matter volume (GMV), (2) white matter volume (WMV), and (3) volumetric symmetry. We hypothesized that chronic LH stroke would be accompanied by significant reductions in cerebellar gray and white matter volume, particularly in the contralesional hemisphere of the cerebellum, with the extent of these cerebellar changes being associated with severity of reported clinical impairments (i.e., aphasia severity) as well as clinical prognosis (i.e., recovery of language abilities following treatment).

**Table 1.** Control and chronic stroke population demographics.

	Chronic stroke (*N* = 249)	Healthy controls (*N* = 244)
Age (yr)	*M* = 60.5, *SD* = 11.4 (29, 81)	*M* = 45.8, *SD* = 11.4 (20, 79)
Gender	Female = 153 (61%)	Female = 185 (76%)
	Male = 96 (39%)	Male = 59 (24%)
Race	White = 208 (84%)	White = 208 (85%)
	Black = 39 (15%)	Black = 25 (10%)
	Other = (1%)	Other = 11 (5%)
Lesion size (cm^3^)	*M* =118.3, *SD* = 96.6 (1.5, 479.2)	N/A
Time post-stroke (days)	*M* = 1639, *SD* = 1672 (205, 9301)	N/A

*Note*. *M* = mean, *SD* = standard deviation. Comma-delimited numbers in parentheses denote minimum and maximum values for each variable.

## MATERIALS AND METHODS

### Participants

Participants included in the following analyses were enrolled over various studies from the Center for the Study of Aphasia Recovery (C-STAR). Chronic LH stroke participants were recruited between the ages of 21 and 80, and were considered eligible for study after 12 months post-onset of an ischemic or hemorrhagic stroke. Individuals excluded from participation include those presenting with contraindications of MRI, bilateral or right hemisphere (RH) stroke, or pre-existing major neurological disorders (such as Parkinson’s or multiple sclerosis). Information for healthy participants was drawn from the University of South Carolina’s Aging Brain Cohort repository (ABC@USC), which is described in a separate paper (Newman-Norlund et al., 2021). Written informed consent was obtained from all individual participants included in the study. This study was approved by the University of South Carolina’s Institutional Review Board.

### Imaging Data Collection

Neuroimaging data were acquired as part of participation in one of multiple National Institutes of Health funded and/or internally funded grants conducted over a period of 16 years. Participants were included in the present study if their participation involved collection of a high-resolution T1-weighted structural MRI scan. For clinical populations, MRI data were acquired using a Siemens 3T scanner which was upgraded from a Prisma Trio to a Prisma FIT in 2016. Properties of the whole-brain 3D GR\IR sequence used in the clinical sample were as follows: repetition time (TR) = 1,160 ms, inversion time (TI) = 600 ms, echo time (TE) = 4.24 ms, flip angle = 15°, 256 × 256 × 192 isotropic voxels. MRI data in healthy participants were acquired using the Massachusetts General Hospital multi-echo whole-brain MPRAGE T1 sequence with the following parameters: TR 2,530 ms, TI = 1,100 ms, TE = (1.44, 2.9, 4.3, 5.82, 7.28), flip angle = 7°, 256 × 256 × 192 isotropic voxels.

### Image Data Processing

Cerebellar volume and total intracranial volume (TIV) were calculated using high-resolution T1-weighted MPRAGE structural MRI images acquired at the McCausland Center for Brain Imaging at the University of South Carolina. All images were processed using MATLAB (MathWorks, 2022), SPM12 (Version 7781; Penny et al., 2007) and the Cat12 (Version 12.6, 1700) VBM toolbox with default settings (Gaser et al., 2022). For individuals with stroke, T1-weighted structural images were enantiomorphically healed prior to processing to ensure accurate normalization (Nachev et al., 2008; Yourganov et al., 2017; see the Imaging section of Supplementary Methods in the Supporting Information, available at https://doi.org/10.1162/nol_a_00120). For the purpose of this paper, cerebellar structural integrity was operationalized as three separate measures: cerebellar GMV, cerebellar WMV, and cerebellar volumetric (both gray and white matter based) lateral symmetry (i.e., left vs. right). Cerebellar volume metrics were derived from each participant using the Cat12 default processing pipeline and normalized by participant’s TIV. This normalization procedure effectively transformed the data from cubic centimeters to percent total brain volume, a unitless measure that accounts for differences in total intracranial volume among participants (Whitwell et al., 2001). Percent total brain volume and symmetry measures (see equation below) were used in subsequent statistical analyses reported in this paper. Gray and white matter percent volumes were computed for the left and right cerebellar hemispheres independently, as well as for each of the 34 (17 LH and 17 RH) spatially distinct regions comprising the SUIT cerebellar atlas (Diedrichsen et al., 2009). For the purpose of this paper, we refer to these derived measures, which represent percent total brain volume (see above), simply as GMV and WMV. Cerebellar asymmetry indices for both GMV and WMV were calculated based on the percent gray and white matter volume described above, using the following standard laterality index (LI) equation (Seghier, 2008):

$$\text{GMV}\;LI=\left(\%\text{Right}\;CV-\%\text{Left}\;CV\right)/\left(\%\text{Right}\;CV+\%\text{Left}\;CV\right)$$

$$\text{WMV}\;LI=\left(\%\text{Right}\;CV-\%\text{Left}\;CV\right)/\left(\%\text{Right}\;CV+\%\text{Left}\;CV\right)$$

### Behavioral Data

Behavioral data included performance on the Western Aphasia Battery—Revised (WAB-R) and the Treated40 object naming task (acquired at two different timepoints: at the time of enrollment and then 6 months following participants’ last study intervention session) were acquired as part of C-STAR, which is comprised of data from research studies conducted between 2006 and 2022. The Treated40 variable represents treatment-related gains recorded from participants in various C-STAR intervention studies (*N* = 77). The Treated40 variable was computed by comparing performance on a 40-item picture naming task before and 6 months after a 5-week aphasia treatment that involved both semantic and phonological intervention, with higher numbers indicating greater pre–post improvement, as follows:

$$\left[\#\;\text{pictures}\;\text{correctly}\;\text{identified}\;\text{after}\;\text{treatment}\right]-\left[\#\;\text{of}\;\text{pictures}\;\text{identified}\;\text{before}\;\text{treatment}\right]$$

Additional details regarding the Treated40 task, its administration, and its properties can be found in Fridriksson et al. (2009; also see Language Measures section of Supplementary Methods).

## RESULTS

**Figure 1.**
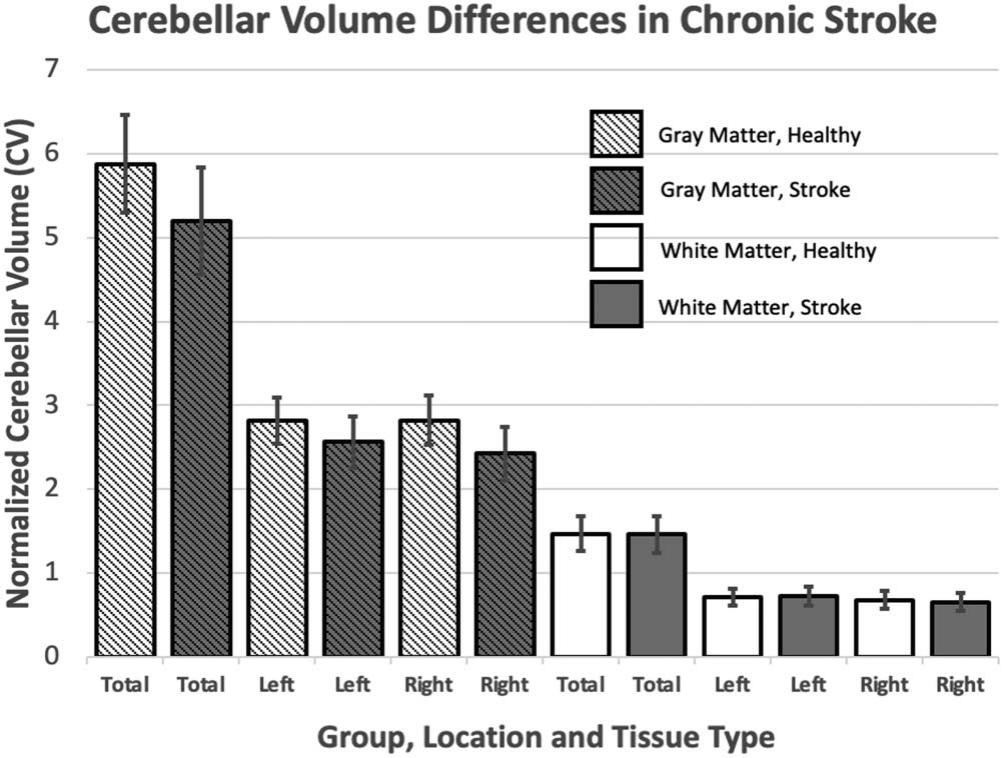
Cerebellar volume in chronic left hemisphere (LH) stroke with aphasia and controls after accounting for age, race, and gender. Cerebellar volume was significantly lower in chronic LH stroke with aphasia and controls in *all areas shown* (right white matter volume, *p* = 0.32, all other areas *p* < 0.001). *Error bars represent standard deviation.

**Figure 2.**
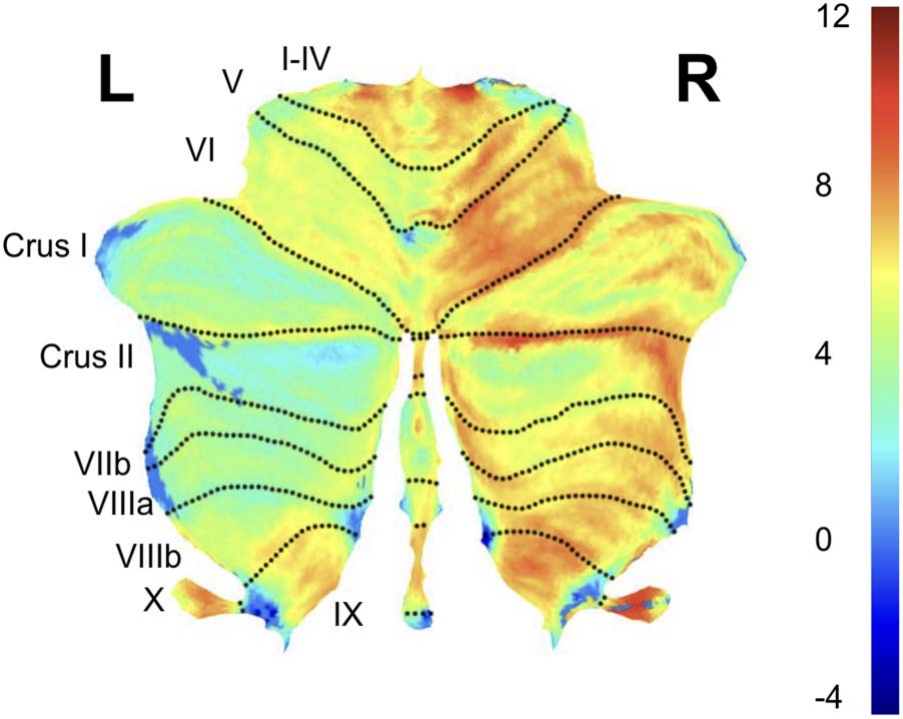
Spatial locations of significant differences in cerebellar volume (controls vs. chronic LH stroke) on a flat-map of the cerebellum created using the SUIT processing suite. Values in colorbar represent spmT values from a direct comparison (independent samples *t* test) of cerebellar gray matter volume (GMV) maps created for each group by SUIT. Differences in cerebellar GMV were most pronounced in the right hemisphere of the cerebellum for chronic LH stroke. These effects were evident across the majority of cerebellar regions (see Supplementary Table 2A).

Based on our hypothesis that cerebellar volume would differ between the healthy and chronic LH stroke groups, we first conducted an uncorrected two-tailed independent samples *t* test for mean differences in total cerebellar volume (GMV + WMV) between the chronic LH group (*M* = 6.67% TIV, *SD* = 0.70% TIV) and the healthy control group (*M* = 7.35% TIV, *SD* = 0.70% TIV). The difference between total cerebellar volume in these groups was significant, *t*(490) = 10.90, *p* < 0.001. We subsequently tested for more specific differences in mean GMV and WMV in left and right hemispheres using the same approach (i.e., uncorrected independent samples *t* tests). Cerebellar GMV in the left hemisphere was different between the chronic LH stroke (*M* = 2.56% TIV, *SD* = 0.30% TIV) and healthy control group (*M* = 2.81% TIV, *SD* = 0.27% TIV), *t*(490) = 9.73, *p* < 0.001. Similarly, cerebellar GMV in the right hemisphere was different between the chronic LH stroke (*M* = 2.43% TIV, *SD* = 0.32% TIV) and healthy control group (*M* = 2.83% TIV, *SD* = 0.29% TIV), *t*(490) = 14.54, *p* < 0.001. Although cerebellar WMV in the left hemisphere did not significantly differ between the chronic LH stroke (*M* = 0.71% TIV, *SD* = 0.10% TIV) and control group (*M* = 0.73 % TIV, *SD* = 0.11% TIV), *t*(490) = −1.28, *p* = 0.20, cerebellar WMV did differ between the chronic LH stroke groups (*M* = 0.66 % TIV, *SD* = 0.10% TIV) and healthy control group (*M* = 0.68 % TIV, *SD* = 0.11% TIV), in the right hemisphere, *t*(490) = 2.21, *p* = 0.028 (Figure 1). As a final, even more fine-grained analysis, we performed a series of independent samples *t* tests across anatomical regions of the cerebellum to directly compare regional cerebellar volume (both GMV and WMV) and regional volumetric asymmetry in the two groups, applying Bonferroni correction using an alpha value of 0.05 (i.e., only *p* < 0.001 was considered significant). Regions were extracted from the SUIT atlas (32 areas; see Figure 2 and Supplementary Tables 2A and 2B).

We further hypothesized that cerebellar laterality would be different in healthy and chronic LH stroke group. To test this, we conducted a pair of two-tailed independent sample *t* tests which revealed that both GMV LI and WMV LI were significantly different in the two groups (*t*(491) = −11.25, *p* < 0.01 and *t*(491) = −6.44, *p* < 0.001), indicating greater asymmetry favoring the left hemisphere in the chronic LH stroke group. These differences were widespread, including all but one subregion of the cerebellum (Vermis Crus I; see Figure 2).

In order to rule out the possibility that changes in cerebellar GMV observed in the stroke group were related solely to age, we examined the relationship between age and cerebellar volume in healthy adults. Consistent with the idea that brain volume generally decreases steadily after age 40 (Peters, 2006), a Pearson correlation revealed that, within the healthy group, increased age was associated with decreased cerebellar GMV (*p* values < 0.001). Importantly, increasing age was not associated with changes in measures of volumetric asymmetry (i.e., LI GMV or the LI WMV, *p* values > 0.05), suggesting that the asymmetry observed in the chronic LH stroke group was likely unrelated to the normal aging process.

Based on the idea that changes in cerebellar GMV resulted from the cortical lesion itself, an assumption buttressed by the finding that changes in cerebellar asymmetry were not associated with aging per se (see above), we next examined correlations between lesion size, cerebellar volume and cerebellar asymmetry. Within the stroke group, a partial Pearson’s correlation controlling for age, gender, and race revealed that lesion size in the left cortical hemisphere, was positively correlated with right cerebellar GMV, *r*(236) = −0.21, *p* < 0.001, but not with left cerebellar GMV (*p* = 0.36). Additionally, lesion size was positively correlated with both LI GMV, *r*(239) = 0.379, *p* < 0.001 and LI WMV, *r*(239) = 0.22, *p* < 0.001 values. Further, we explored the possibility that changes in cerebellar volume were related to time since stroke by conducting a Pearson correlation between cerebellar volume, asymmetry measures, and days post-stroke (at the time of the MRI scan). Days post-stroke was positively correlated with both LI GMV, *r*(239) = 0.19, *p* < 0.005, and LI WMV, *r*(239) = 0.12, *p* < 0.05 scores, meaning that cerebellar volumetric imbalance increased as a function of time (Supplementary Table 2A and Table 2B). After controlling for lesion size, age, race, and sex, a two-tailed partial correlation between days post-stroke and cerebellar GMV LI remained significant, *r*(240) = 0.132, *p* = 0.042, while the correlation with WMV LI was no longer significant, *r*(240) = 0.155, *p* = 0.16.

**Figure 3.**
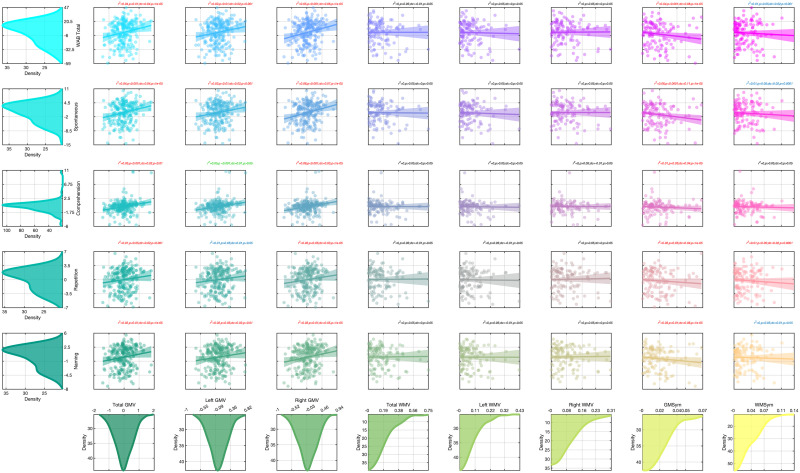
One-sample partial Pearson and partial distance (i.e., nonlinear) correlations between WAB scores (rows) and cerebellar volume/symmetry indices (columns) after conditioning on age, sex, race, lesion size, and days since stroke. Red text above each scatterplot highlights relationships significant for both types of correlations (*p* < 0.05). Black text indicates relationship insignificance for both types of correlations. Blue text indicates significant nonlinear relationship and insignificant linear relationship. Green text indicates significant linear relationship and insignificant nonlinear relationship. *r*^2^ = partial Pearson correlation coefficient squared (i.e., explained variance), DC = distance correlation coefficient, GVM = gray matter volume, WMV = white matter volume, WAB = Western Aphasia Battery, Spontaneous = spontaneous speech subscore, Comp = comprehension subscore, Repetition = repetition subscore, Naming = naming subscore.

In general, there was a strong correlation between global measures of cerebellar GMV, LI GMV, and WAB-R scores. Notably a set of two planned Pearson correlations revealed that overall, left and right cerebellar GMV and WMV were positively correlated with WAB_TOTAL_ scores (all *p* values < 0.001). Moreover, the positive correlation between cerebellar GMV and WMV and language ability was significant for each individual WAB-R subscore (all *p* values ≦ 0.05). Relationships between these variables that survived conditioning on age, sex, race, lesion size, and days post-stroke are shown in Figure 3.

In order to assess the value of adding region-specific cerebellar GMV to quantitative models of language ability (i.e., WAB_TOTAL_), above and beyond other available metrics, we conducted a one-way analysis of variance (ANOVA) comparing two separate linear regression models. The first model used WAB_TOTAL_ as the dependent variable with demographic and cortical lesion characteristics as the independent variables, while the second model added cerebellar GMV values (one for each SUIT atlas region of interest [ROI]) as predictors. Addition of cerebellar GMV data to the model significantly improved fit, with an adjusted R^2^ change from H_0_ = 0.41, to H_1_ = 0.31, 54 *F*(34, 76) = 7.15, *p* < 0.001 (Supplementary Table 4). Specific SUIT atlas regions in which greater cerebellar GMV was associated with improved scores included left VI (*p* = 0.025), right VIIa (*p* = 0.009), and vermis VIIb (*p* = 0.10). In order to further explore the value of adding cerebellar GMV data to models predicting specific linguistic abilities, we conducted a series of additional one-way ANOVAs as described above, this time treating each of the four WAB subscores {spontaneous speech, repetition, comprehension, naming} as the dependent variable. In all cases, addition of cerebellar GMV data to linear regression models significantly improved model fit (all *p* values < 0.001) above and beyond models that included only demographic and cortical lesion data. These models identified a number of cerebellar regions in which higher GMV was associated with higher WAB-R subscores. Higher spontaneous speech subscores were associated with greater GMV in left I-IV (*p* = 0.049), Vermis VIIb (*p* = 0.010), right VIIIa (*p* = 0.002), and right VIIIb (*p* = 0.01); higher naming subscores were associated with greater GMV in left VI (*p* = 0.039) and right VIIIa (*p* = 0.038); and higher repetition subscores were associated with greater GMV in left VI (*p* = 0.027), Vermis VIIb (*p* = 0.023), and right VIIIa (*p* = 0.006). No regions emerged from the analysis of comprehension subscores (all *p*s < 0.08). Notably, both total GMV and GMV asymmetry were highly correlated with WAB_TOTAL_ scores, *r*(232) = 0.21, *p* < 0.001.

**Figure 4.**
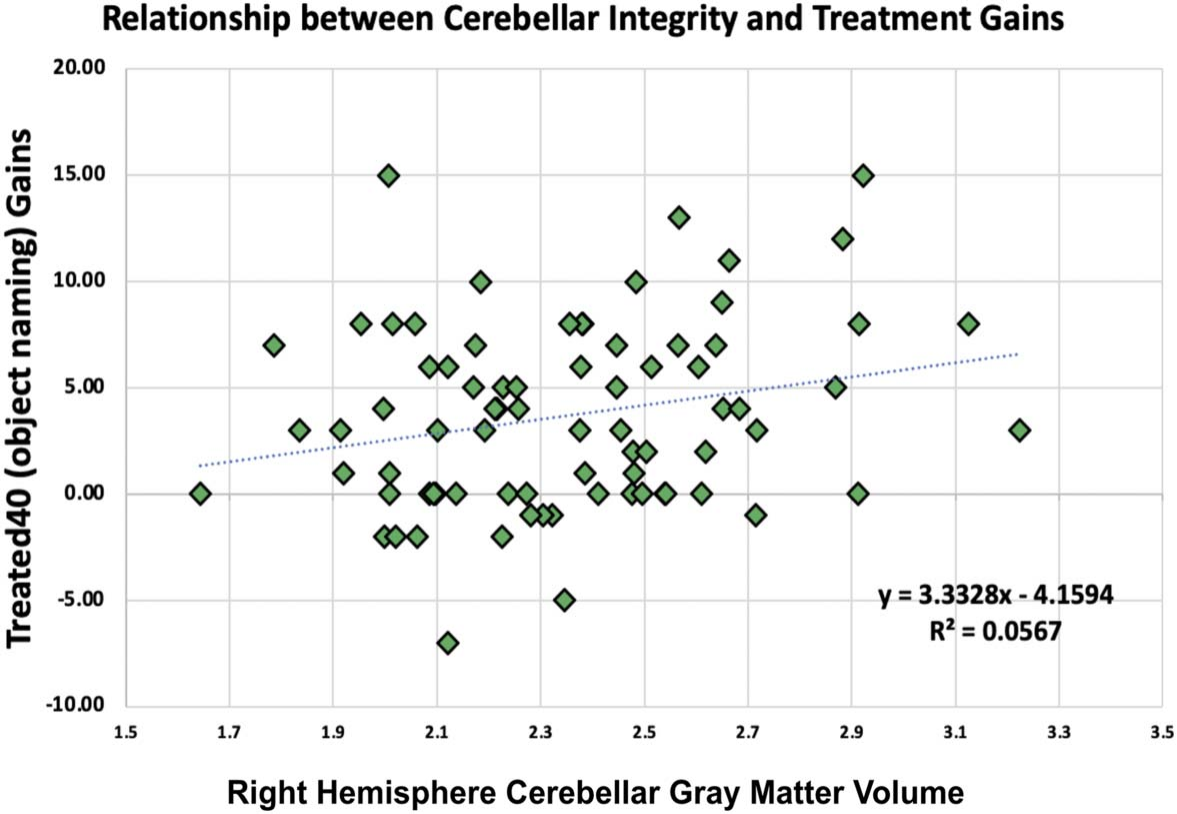
Within our sample of chronic LH stroke, a number of participants took part in aphasia treatment studies (*N* = 77) involving both phonological and semantic training. We found that right cerebellar GMV accounted for significant additional variability in treatment gains, as defined by changes in *Treated40* (i.e., improvement in ability to name visually cued objects) scores. Cerebellar GMV was predictive of improvements even after accounting for demographic (age, race, gender) and cortical lesion (lesion size and days post-stroke) characteristics.

Finally, we assessed the value of adding region-specific cerebellar GMV data to regression models of treatment-related gains in picture naming ability. Specifically, we conducted a one-way ANOVA comparing two separate linear regression models using treatment-related gains as the dependent variable (i.e., difference in performance on a picture-cued object naming task compared both before and after treatment; see Supplementary Methods). As with the models described above, used to assess the relationship between WAB-R scores and region-specific cerebellar GMV, the first model was constrained to using demographic variables and lesion characteristics, whereas the second model additionally included region-specific cerebellar GMV data. Comparison of both models demonstrated that the model which included region-specific cerebellar GMV values was significantly better than the model using demographic and cortical lesion measures alone (adjusted R^2^ change from H_0_ = 0.15 to H_1_ = 0.31, *F*(34, 76) = 2.001, *p* = 0.016, indicating that participants with greater cerebellar GMV experienced greater treatment gains; Figure 4). This effect was driven by cerebellar GMV differences in right cerebellar area Va (*p* = 0.003; Supplementary Table 4).

## DISCUSSION

Cortical stroke is associated with both functional and structural changes at sites distal from the area of original infarct, and this diaschisis has been a primary focus of researchers and clinicians interested in predicting the severity of post-stroke sequelae as well as the likelihood of future recovery (Baldassarre et al., 2016; Kalinosky et al., 2017; Tang et al., 2016; Wilmskoetter et al., 2019, 2021). Cortical damage also impacts the cerebellum, via crossed cerebrocortical connections, but less is known about the impact these changes have on stroke-related impairments. By examining cerebellar morphometry/asymmetry in individuals with aphasia as a consequence of chronic LH stroke, we were able to probe the relationship between cerebellar integrity (operationalized as GMV and asymmetry), language ability, and treatment response. This study is the first and largest to demonstrate clear and clinically meaningful changes in the contralateral cerebellar cortex in chronic LH stroke. We show that the inclusion of cerebellar GMV and symmetry metrics significantly improves models of language ability (WAB-R scores) and improvement trajectory (changes in picture naming), above and beyond models based on demographic and cortical lesion-related predictors alone.

### Cortical Lesion Characteristics and Cerebellar GMV Changes

Within our chronic LH stroke cohort, we observed a strong positive correlation between lesion size and changes in cerebellar GMV and laterality. Notably, these changes were not present in a healthy cohort, even after controlling for demographic factors such as age, gender, and race, suggesting that the observed changes were not simply the product of age-related central nervous system (CNS) atrophy. We also found that cerebellar imbalance, operationalized as asymmetry between left and right cerebellar GMV, was positively correlated with days post-stroke, a finding that held true even when controlling for demographic factors (age, race, sex) and lesion size. This finding raises the possibility that changes in cerebellar volume may still occur well into the chronic stages of stroke, a possibility which has not previously been considered. While more research is clearly necessary both to confirm this, and, if confirmed, to fully delineate the time course of cerebellar GMV changes following stroke, the possibility that such changes may occur over a period of years could have profound implications for aphasia treatment. Most importantly, exploring this relationship could help to define the therapeutic time window during which potentially ameliorative/preventative adjuvant therapies are maximally beneficial.

### Cerebellar GMV Changes in Chronic LH Stroke

A striking aspect of this study is the highly significant and widespread nature of the changes in cerebellar GMV and gray matter symmetry observed in individuals with chronic LH stroke. Compared to healthy adults, individuals with chronic LH stroke showed significantly lower cerebellar GMV in both the left and right cerebellar hemispheres, and this difference was particularly apparent within right cerebellar gray matter. The significant differences in the laterality of cerebellar GMV in individuals with chronic LH stroke, argues against concern that changes in cerebellar GMV are simply the result of age-related global CNS atrophy. This finding is not particularly surprising, given prior research describing structural connectivity between the left cerebral hemisphere and the right cerebellum (Diedrichsen et al., 2009; Jansen et al., 2005; Moberget & Ivry, 2016).

The most parsimonious explanation for reductions in cerebellar GMV observed in the chronic LH group in the current study is that it resulted from the process of Wallerian (anterograde) degeneration, a process that occurs when cerebellar cell density is decreased following removal of input from cortical cells. Based on what is known about this process, downstream effects on the cerebellum GMV would be expected to evolve over the course of weeks or months. For example, necrosis of cortical neurons, caused by stroke-related hypoperfusion or hemorrhage, typically takes place within a timeframe of days to weeks, with survival of perilesional neurons likely varying as a function of their distance from the primary lesion site (Brown, 2021). Following death of these cortical cells, the survival of contralateral cerebellar cells denied their input would likely depend on several factors, including the number of connections removed, remaining connectivity, and time since injury, all of which are difficult to measure but would likely evolve across weeks as well (Fink & Cookson, 2005). One intriguing possibility is changes in the cortex result in changes within the cerebellum that, in turn, result in further cortical changes. That the existence of this decidedly “vicious cycle” could continue to alter brain structure for months or even years after stroke is certainly plausible. Indeed, there is already evidence that stroke lesions continue to expand years after stroke (Seghier et al., 2014). However, it is also clear that more research must be done to chart the exact time course of this process and the exact mechanisms supporting these degenerative cerebrocortical effects.

### Aphasia Severity and Cerebellar GMV in Chronic LH Stroke

An important finding of the current study is that cerebellar GMV and asymmetry have clinical significance. Cerebellar GMV, WMV (left hemisphere, right hemisphere, and total volume) and asymmetry were positively correlated with each of the WAB-R subscores (spontaneous speech, repetition, naming, and comprehension). These broad measures of cerebellar health were significant predictors of WAB-R scores even after controlling for demographic (age, race, sex) and cortical putative lesion-based measures (lesion size, days post-stroke), traditionally thought to account for the greater part of variability in chronic stroke aphasia severity and treatment outcomes (Schiemanck et al., 2005; Figure 3).

Regression models of both aphasia severity (as measured by the WAB-R) and treatment recovery (as measured by improvements in picture-cued object naming) benefitted from inclusion of cerebellar GMV data. Using an ROI based analysis in which the cerebellum was divided into 34 spatially distinct areas, we demonstrate that regional cerebellar GMV explains unique variability in both aphasia severity and treatment trajectories, significantly improving models based on other known factors. This finding was also illustrated in a prior meta-analysis identifying cerebellar areas Crus I and Crus II, as well as Lobules VI and VIIA (Mariën & Manto, 2015). Our results, which identify a number of specific cerebellar ROIs in which WAB-R and subscores were significantly correlated with higher cerebellar GMV including left 1-IV, left VI, right VIIIa, right VIIIb, and Vermis VIIb partially overlap with these findings. For example, our data indicated that higher cerebellar GMV in left area VI was associated with higher WAB-R_TOTAL_ scores as well as higher scores on the repetition and naming WAB-R subscores. Interestingly, we also found an association between WAB-R spontaneous speech scores and GMV in two areas that have been linked to working memory, namely left area I-IV (Guell et al., 2018) and right area VIIIa (Stoodley, 2014; Stoodley & Schmahmann, 2009). This makes sense given the fact that working memory is known to be important for retrieval of words and construction of more complex, multiword utterances (Baddeley, 2003; Deldar et al., 2021). Although not identified in the meta-analysis by Mariën and Manto (2015), right cerebellar regions VIIb and VIIIa have been linked to linguistic functions by other researchers (Stoodley & Schmahmann, 2009), with area VIIb showing increased fMRI signal during a verb generation task (Stoodley et al., 2012), and one study reported that higher cerebellar volumes in cerebellum area VIIIa was linked to higher language abilities in children with autism spectrum disorder (Hodge et al., 2010).

### Aphasia Treatment Response and Cerebellar GMV in Chronic LH Stroke

An important finding in the current study was that inclusion of GMV in linear regression models of treatment response significantly improved model fit, increasing variance from R2 = 0.21 to R2 = 0.62, a change of 0.41, with a calculated effect size of 1.62, Cohen’s f2 = R2/1 − R2/(DF_MODEL_ − DF_RESIDUAL_). Cerebellar GMV within right area V was the most significant predictor in this model. Right cerebellar area V has been implicated in both working memory and emotional processing. Taken together with findings, described above, that also implicate working memory related areas in aphasia severity, this finding provides compelling converging evidence that working memory is a key factor in treatment response in chronic LH aphasia. This meshes well with prior work investigating working memory and language ability in aphasia. For example, according to a study by Lee and Pyun (2014), attention and working memory were significantly impaired in aphasic patients compared to controls. In another study, aphasia severity and working memory impairments were highly correlated (Yu et al., 2013). There is also some evidence that treatments designed to rehabilitate short-term and working memory can improve language function following aphasia (Majerus, 2018; Martin et al., 2021).

One of the main clinical payoffs of precisely localizing cerebellar changes associated with language and stroke is that such findings can inform targeting procedures of brain-stimulation based treatments. Indeed, the impact of cerebellar stimulation, specifically transcranial direct current stimulation (tDCS), has already been investigated within the field of stroke, with some initial studies showing that application of deep brain stimulation and tDCS to the cerebellum can improve recovery in both animal and human models, possibly by enhancing the excitability of surviving cortical neurons (Machado & Baker, 2012; Sebastian et al., 2017; van Dun & Manto, 2018). Critically, both the optimal timing of brain stimulation based approaches, as well as the exact dosages needed to maximize recovery remain unknown. These factors will have to be considered in relation to the evolution of cortical and cerebellar changes following stroke, which is temporally complex and likely evolves in ways that are not currently well understood. Both downstream and upstream pathways need to be considered based on the known relationship between cerebral and cerebellar function (i.e., although beyond the scope of this paper, extant literature suggests that, in addition to cerebellar stimulation, cortical stimulation may positively impact recovery from aphasia following stroke; Ashaie et al., 2022). The optimization of stimulation location and treatment timing presents a clear avenue for future research and an important opportunity for collaboration between clinicians and researchers working with both acute and chronic stroke populations.

The interpretation of this study is necessarily subject to certain limitations. First, the current sample was limited to a relatively homogeneous cohort of individuals with LH stroke with aphasia, making it uncertain whether or not the patterns identified here will hold for other types of stroke or stroke-related sequalae. Second, ROI-related overlap between findings from the present study and prior investigations of the relationship between language and the cerebellum (using healthy or other clinical populations) should be interpreted with caution given the known potential for neural reorganization following stroke. An additional limitation, albeit one that presents a tantalizing opportunity for future investigation, is that the present study did not attempt to relate specific patterns of cortical damage to specific patterns of change in cerebellar GMV or laterality. Cortical models of aphasia severity and recovery that account for the spatial distribution of the lesion, in addition to lesion size, are generally superior (Forkel & Catani, 2018; Fridriksson, 2010; Park et al., 2016; Sul et al., 2019). If spatial characteristics of cortical lesions are meaningfully related to changes observed in the cerebellum, then it may be possible to further improve the fit of predictive models of aphasia severity and/or treatment response by combining these data.

## GENERAL CONCLUSION

The current study highlights the importance of cerebellar integrity in individuals with chronic LH stroke and aphasia. By identifying differences in cerebellar GVM in healthy individuals and individuals with stroke, this study demonstrates that cortical stroke has a significant impact on cerebellar volume and symmetry. An attempt to understand the implications of these novel findings also raises interesting new questions about the nature of neural changes following stroke. For example, What is the time window over which cortical and cerebellar changes occur? How are cortical and cerebellar changes linked together [do they reverberate]? What is the relationship between spatial characteristics of cortical lesions and spatial properties of cerebellar GMV changes? And, finally, How can we use the answers to all of these questions to inform the design of better brain stimulation based treatment interventions for chronic LH stroke and aphasia, or any other type of stroke and subsequent sequelae for that matter? Clearly, these questions would best be answered by a comprehensive longitudinal research program in which experts in acute and chronic stroke collaborate to investigate cerebral and cerebellar changes as they evolve across time. In the end, pursuit of such research promises plenty of potential gains for researchers interested in brain plasticity, clinicians interested in improving aphasia-related impairments, and patients dedicated to recovery and discovery.

## FUNDING INFORMATION

Julius Fridriksson, National Institutes of Health, Award ID: P50DC014664. Julius Fridriksson, National Institutes of Health, Award ID: U01DC011739. Julius Fridriksson, National Institutes of Health, Award ID: R01DC008355. Julius Fridriksson, National Institutes of Health, Award ID: P50DC014664.

## AUTHOR CONTRIBUTIONS

**Roger D. Newman-Norlund**: Conceptualization: Lead; Formal analysis: Lead; Investigation: Lead; Project administration: Lead; Visualization: Lead; Writing – original draft: Lead; Writing – review & editing: Lead. **Makayla Gibson**: Conceptualization: Supporting; Methodology: Supporting; Writing – original draft: Supporting; Writing – review & editing: Supporting. **Lisa Johnson**: Visualization: Supporting; Writing – review & editing: Supporting. **Alex Teghipco**: Formal analysis: Supporting; Visualization: Supporting; Writing – review & editing: Supporting. **Chris Rorden**: Funding acquisition: Supporting; Visualization: Supporting; Writing – review & editing: Supporting. **Leonardo Bonilha**: Writing – review & editing: Supporting. **Julius Fridriksson**: Funding acquisition: Lead; Investigation: Supporting; Resources: Equal; Writing – review & editing: Supporting.

## DATA AND CODE AVAILABILITY STATEMENT

The data sets generated and/or analyzed during the current study are available from the corresponding author upon request. All code used is publicly available or available upon request.

## Supplementary Material



## TECHNICAL TERM

Diaschisis: A sudden change of function in a portion of the brain connected to a distant, but damaged, brain area.
