# Supplementary material for: Cerebellar Atrophy and Language Processing in Chronic Left-Hemisphere Stroke
Source: Neurobiol Lang (Camb). 2024 Aug 15;5(3):722–35. doi: 10.1162/nol_a_00120 (PMC11338304; doi:10.1162/nol_a_00120)
Supplement: Supplementary file 1 [file nol-5-3-722-s001.pdf]

Supplementary Table 1A. Correlations between demographic factors and cerebellar volume in healthy adult participants. Correlations where 0.12 < *r* > –0.12 are significant at $p < 0.05$.

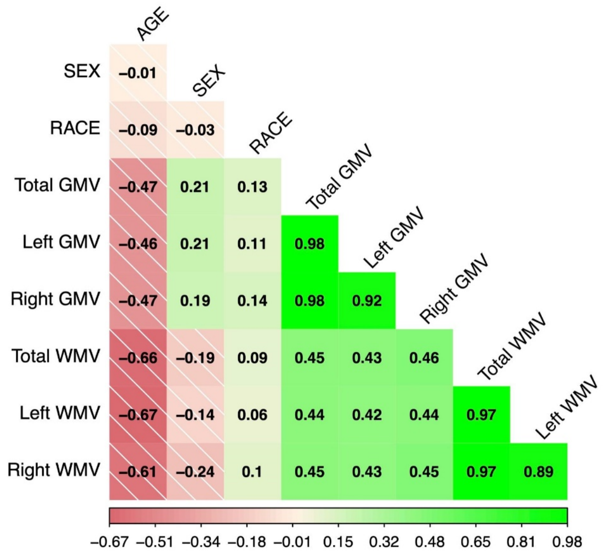

Formatted: Font: Bold

Deleted: -

Deleted: ,

Formatted: Font: Italic

Formatted: Font: Italic

Deleted: ¶
¶

Supplementary Table 1B. Correlations between demographic factors and cerebellar volume in individuals with chronic LH stroke. Correlations where 0.12 < *r* > –0.12 are significant at $p < 0.05$.

Formatted: Font: Bold

Deleted: -

Deleted: ,

Formatted: Font: Italic

Formatted: Font: Italic

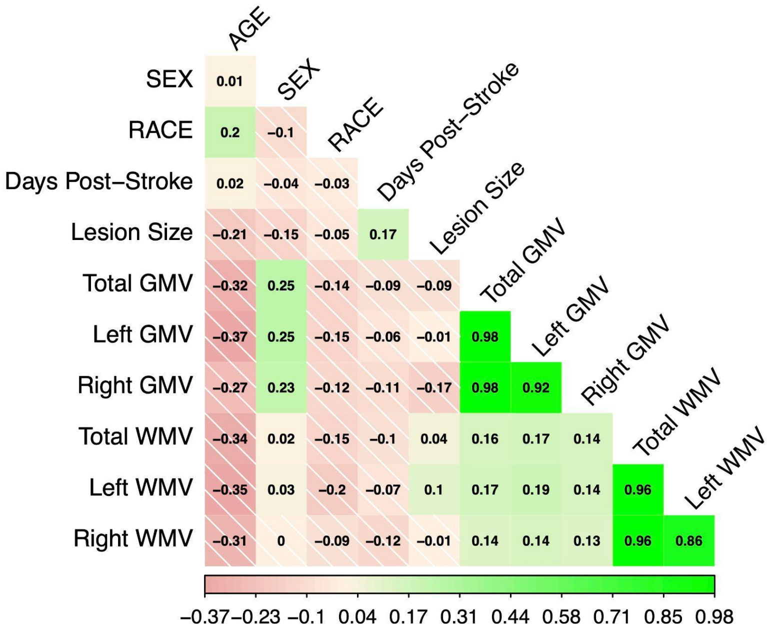

Deleted: ¶
¶
¶

Supplementary Table 2A. Differences in cerebellar GMV in healthy and stroke groups, shown separately for each individual cerebellar gray matter region in the SUIT atlas, after controlling for differences in age, race, and gender.

Area	type	N	Mean	min	max	stDev	SEM	t	df	p-value	effect size
**Left_I-IV**	heathy	244	0.1308	0.085	0.089	0.015	0.0009	6.122	491	<.001	0.546
	stroke	249	0.1218	0.119	0.0789	0.018	0.0011				
**Right_I-IV**	heathy	244	0.1559	0.096	0.1084	0.018	0.0011	7.717	491	<.001	0.688
	stroke	249	0.1426	0.121	0.0959	0.021	0.0013				
**Left_V**	heathy	244	0.1918	0.12	0.131	0.02	0.0013	8.447	491	<.001	0.753
	stroke	249	0.1764	0.124	0.1219	0.021	0.0013				
**Right_V**	heathy	244	0.2023	0.164	0.1081	0.023	0.0014	13.303	491	<.001	1.186
	stroke	249	0.1744	0.126	0.1218	0.024	0.0015				
**Left_VI**	heathy	244	0.4558	0.379	0.208	0.053	0.0033	12.026	491	<.001	1.072
	stroke	249	0.3994	0.306	0.2676	0.052	0.0033				
**Vermis_VI**	heathy	244	0.0793	0.054	0.0558	0.01	0.0006	7.225	491	<.001	0.644
	stroke	249	0.073	0.06	0.0508	0.01	0.0007				
**Right_VI**	heathy	244	0.4308	0.414	0.1581	0.051	0.0032	16.08	491	<.001	1.434
	stroke	249	0.3574	0.261	0.25	0.051	0.0032				
**Left_CrusI**	heathy	244	0.6593	0.466	0.3994	0.081	0.0051	9.323	491	<.001	0.831
	stroke	249	0.5918	0.499	0.3805	0.082	0.0051				
**Vermis_CrusI**	heathy	244	0.0006	0.002	0	3E-04	0	0.897	491	0.37	0.08
	stroke	249	0.0006	0.002	0	3E-04	0				
**Right_CrusI**	heathy	244	0.6399	0.625	0.2078	0.081	0.0051	12.077	491	<.001	1.077
	stroke	249	0.5519	0.516	0.3304	0.082	0.0052				
**Left_CrusII**	heathy	244	0.4895	0.346	0.3127	0.06	0.0038	6.878	491	<.001	0.613
	stroke	249	0.4514	0.367	0.3069	0.064	0.004				
**Vermis_CrusII**	heathy	244	0.0191	0.016	0.0103	0.003	0.0002	4.622	491	<.001	0.412
	stroke	249	0.018	0.016	0.0114	0.003	0.0002				
**Right_CrusII**	heathy	244	0.482	0.534	0.1903	0.063	0.004	12.13	491	<.001	1.082
	stroke	249	0.411	0.379	0.2303	0.068	0.0043				
**Left_VIIb**	heathy	244	0.2734	0.204	0.1797	0.034	0.0021	6.557	491	<.001	0.585
	stroke	249	0.2519	0.201	0.1644	0.039	0.0025				
**Vermis_VIIb**	heathy	244	0.0096	0.012	0.0047	0.002	0.0001	11.461	491	<.001	1.022
	stroke	249	0.0079	0.01	0.0043	0.002	0.0001				
**Right_VIIb**	heathy	244	0.2739	0.299	0.0969	0.036	0.0023	12.962	491	<.001	1.156
	stroke	249	0.2304	0.255	0.1186	0.039	0.0025				
**Left_VIIIa**	heathy	244	0.2602	0.26	0.1124	0.03	0.0019	6.554	491	<.001	0.584
	stroke	249	0.2414	0.181	0.1621	0.035	0.0022				
**Vermis_VIIIa**	heathy	244	0.0494	0.035	0.0305	0.006	0.0004	7.298	491	<.001	0.651
	stroke	249	0.0452	0.046	0.0234	0.007	0.0004				
**Right_VIIIa**	heathy	244	0.2534	0.245	0.0915	0.03	0.0019	12.427	491	<.001	1.108
	stroke	249	0.2168	0.229	0.1364	0.036	0.0022				
**Left_VIIIb**	heathy	244	0.1908	0.188	0.0826	0.025	0.0015	4.871	491	<.001	0.434
	stroke	249	0.1796	0.167	0.1054	0.027	0.0017				
**Vermis_VIIIb**	heathy	244	0.0274	0.023	0.0156	0.004	0.0003	7.013	491	<.001	0.625
	stroke	249	0.0248	0.029	0.0152	0.004	0.0003				
**Right_VIIIb**	heathy	244	0.1964	0.18	0.0918	0.025	0.0016	10.016	491	<.001	0.893
	stroke	249	0.1729	0.184	0.0844	0.028	0.0017				
**Left_IX**	heathy	244	0.1373	0.132	0.0736	0.023	0.0014	7.119	491	<.001	0.635
	stroke	249	0.1224	0.124	0.0732	0.024	0.0015				
**Vermis_IX**	heathy	244	0.0341	0.03	0.02	0.006	0.0004	7.707	491	<.001	0.687
	stroke	249	0.03	0.037	0.0139	0.006	0.0004				
**Right_IX**	heathy	244	0.1557	0.15	0.0728	0.023	0.0015	9.441	491	<.001	0.842
	stroke	249	0.1351	0.133	0.0758	0.025	0.0016				
**Left_X**	heathy	244	0.0269	0.024	0.0123	0.004	0.0002	3.871	491	<.001	0.345
	stroke	249	0.0256	0.023	0.0154	0.004	0.0003				
**Vermis_X**	heathy	244	0.0145	0.014	0.0081	0.002	0.0002	6.924	491	<.001	0.617
	stroke	249	0.013	0.016	0.0081	0.002	0.0002				
**Right_X**	heathy	244	0.0292	0.022	0.0182	0.004	0.0002	4.762	491	<.001	0.425
	stroke	249	0.0275	0.023	0.0186	0.004	0.0003				
**Left_Total**	heathy	244	2.8158	1.928	1.8104	0.281	0.0177	9.717	491	<.001	0.866
	stroke	249	2.5617	1.643	1.807	0.305	0.0192				
**Right_Total**	heathy	244	2.8193	2.556	1.159	0.298	0.0188	14.565	491	<.001	1.299
	stroke	249	2.4201	1.717	1.6429	0.317	0.02				
**GMV LI**	heathy	244	-2E-04	0.507	-0.106	0.028	0.0018	-11.35	491	<.001	-1.012
	stroke	249	0.0274	0.17	-0.046	0.026	0.0017				

Formatted: Font: Bold

Deleted: GM

Deleted: ¶

Supplementary Table 2B. Differences in cerebellar WMV in healthy and stroke groups, shown separately for each individual cerebellar white matter region in the SUIT atlas, after controlling for differences in age, race, and gender.

Formatted: Font: Bold

Deleted: WM

	type	N	Mean	min	max	stDev	SEM	t	df	p-value	effect size
**Left_I-IV**	heathy	244	0.0522	0.039	0.0318	0.007	0.0004	-0.708	491	0.479	-0.063
	stroke	249	0.0527	0.055	0.0275	0.008	0.0005				
**Right_I-IV**	heathy	244	0.0562	0.051	0.0314	0.008	0.0005	4.96	491	<.001	0.442
	stroke	249	0.0525	0.058	0.0273	0.009	0.0005				
**Left_V**	heathy	244	0.0701	0.05	0.048	0.009	0.0006	-2.375	491	0.018	-0.212
	stroke	249	0.0723	0.066	0.0387	0.011	0.0007				
**Right_V**	heathy	244	0.054	0.045	0.0342	0.009	0.0006	0.683	491	0.495	0.061
	stroke	249	0.0534	0.056	0.0263	0.01	0.0006				
**Left_VI**	heathy	244	0.125	0.122	0.074	0.023	0.0014	-4.819	491	<.001	-0.43
	stroke	249	0.1354	0.147	0.0799	0.026	0.0016				
**Vermis_VI**	heathy	244	0.025	0.027	0.0135	0.005	0.0003	4.35	491	<.001	0.388
	stroke	249	0.0231	0.03	0.0089	0.005	0.0003				
**Right_VI**	heathy	244	0.0953	0.141	0.0254	0.021	0.0013	-0.91	491	0.363	-0.081
	stroke	249	0.0971	0.123	0.0445	0.023	0.0015				
**Left_CrusI**	heathy	244	0.1446	0.207	0.0849	0.029	0.0018	1.05	491	0.294	0.094
	stroke	249	0.1418	0.218	0.0796	0.031	0.0019				
**Vermis_CrusI**	heathy	244	0	4E-04	0	1E-04	0	0.495	491	0.621	0.044
	stroke	249	0	5E-04	0	1E-04	0				
**Right_CrusI**	heathy	244	0.1531	0.237	0.0261	0.033	0.0021	4.086	491	<.001	0.364
	stroke	249	0.1414	0.193	0.0765	0.031	0.002				
**Left_CrusII**	heathy	244	0.125	0.148	0.0614	0.024	0.0015	-0.455	491	0.649	-0.041
	stroke	249	0.1261	0.148	0.0542	0.026	0.0016				
**Vermis_CrusII**	heathy	244	0.0043	0.007	0.0011	0.001	0.0001	0.286	491	0.775	0.025
	stroke	249	0.0042	0.01	0.0011	0.002	0.0001				
**Right_CrusII**	heathy	244	0.0946	0.14	0.0309	0.023	0.0015	-0.224	491	0.822	-0.02
	stroke	249	0.0951	0.158	0.0333	0.024	0.0015				
**Left_VIIb**	heathy	244	0.0485	0.06	0.0215	0.011	0.0007	-2.673	491	0.008	-0.238
	stroke	249	0.0517	0.086	0.0165	0.015	0.0009				
**Vermis_VIIb**	heathy	244	0.0019	0.004	0.0004	6E-04	0	-3.878	491	<.001	-0.346
	stroke	249	0.0021	0.004	0.0007	6E-04	0				
**Right_VIIb**	heathy	244	0.0604	0.064	0.0321	0.012	0.0007	-4.534	491	<.001	-0.404
	stroke	249	0.0658	0.072	0.0297	0.015	0.0009				
**Left_VIIIa**	heathy	244	0.047	0.062	0.0202	0.011	0.0007	-2.97	491	0.003	-0.265
	stroke	249	0.0506	0.105	0.0134	0.016	0.001				
**Vermis_VIIIa**	heathy	244	0.0252	0.022	0.014	0.005	0.0003	0.379	491	0.705	0.034
	stroke	249	0.025	0.032	0.0095	0.006	0.0003				
**Right_VIIIa**	heathy	244	0.0536	0.06	0.0243	0.012	0.0007	-2.031	491	0.043	-0.181
	stroke	249	0.0561	0.075	0.0218	0.016	0.001				
**Left_VIIIb**	heathy	244	0.0497	0.06	0.0177	0.011	0.0007	2.164	491	0.031	0.193
	stroke	249	0.0473	0.074	0.0172	0.013	0.0008				
**Vermis_VIIIb**	heathy	244	0.0072	0.011	0.0016	0.002	0.0001	-1.138	491	0.255	-0.102
	stroke	249	0.0074	0.014	0.0011	0.002	0.0002				
**Right_VIIIb**	heathy	244	0.0489	0.064	0.021	0.012	0.0008	5.94	491	<.001	0.53
	stroke	249	0.0422	0.084	0.0109	0.013	0.0008				
**Left_IX**	heathy	244	0.0458	0.055	0.018	0.012	0.0008	4.041	491	<.001	0.36
	stroke	249	0.0413	0.073	0.0081	0.013	0.0008				
**Vermis_IX**	heathy	244	0.0126	0.021	0.0016	0.004	0.0002	-2.328	491	0.02	-0.208
	stroke	249	0.0134	0.024	0.0019	0.004	0.0003				
**Right_IX**	heathy	244	0.0526	0.07	0.0192	0.014	0.0008	6.483	491	<.001	0.578
	stroke	249	0.0445	0.087	0.0074	0.015	0.0009				
**Left_X**	heathy	244	0.0075	0.008	0.0044	0.001	0.0001	1.492	491	0.136	0.133
	stroke	249	0.0073	0.01	0.0039	0.002	0.0001				
**Vermis_X**	heathy	244	0.001	0.003	0	7E-04	0	-5.544	491	<.001	-0.494
	stroke	249	0.0014	0.004	0	9E-04	0.0001				
**Right_X**	heathy	244	0.0071	0.009	0.004	0.002	0.0001	6.08	491	<.001	0.542
	stroke	249	0.0063	0.008	0.0031	0.002	0.0001				
**Left_Total**	heathy	244	0.7154	0.571	0.4349	0.099	0.0062	-1.169	491	0.243	-0.104
	stroke	249	0.7264	0.681	0.4624	0.11	0.007				
**Right_Total**	heathy	244	0.6758	0.654	0.3238	0.107	0.0068	2.3	491	0.022	0.205
	stroke	249	0.6543	0.55	0.4186	0.102	0.0065				
**WMV LI**	heathy	244	0.03	0.37	-0.064	0.037	0.0024	-6.498	491	<.001	-0.579
	stroke	249	0.0526	0.29	-0.092	0.041	0.0026				

Supplementary Table 3. Partial between Western Aphasia Battery (WAB) language scores and cerebellar measures after controlling for controlling for the effects of age, gender, race, lesion size, and days post stroke.

	Total GMV	Left GMV	Right GMV	Total WMV	Left WMV	Right WMV	WAB Total	WAB SponSpeech	WAB Comp	WAB Rep	WAB Naming	GMV Symmetry
Left GMV	0.98											
Right GMV	0.98	0.92										
Total WMV	0.03	0.04	0.03									
Left WMV	0.04	0.05	0.04	0.96								
Right WMV	0.02	0.02	0.02	0.96	0.86							
WAB Total	0.21	0.18	0.23	0	−0.03	0.01						
WAB SponSpeech	0.22	0.18	0.25	−0.02	−0.05	0	0.93					
WAB Comp	0.23	0.23	0.24	0	−0.02	0	0.54	0.4				
WAB Rep	0.14	0.13	0.15	0.02	−0.01	0.04	0.91	0.78	0.41			
WAB Naming	0.17	0.15	0.19	0.02	0.01	0.03	0.92	0.79	0.45	0.84		
GMV Symmetry	−0.26	−0.08	−0.42	−0.05	−0.04	−0.05	−0.18	−0.24	−0.1	−0.1	−0.15	
WMV Symmetry	0.03	0.03	0.03	−0.1	0.16	−0.37	−0.08	−0.09	−0.05	−0.1	−0.05	0.03

Deleted: ¶
Deleted:
Formatted: Font: Bold
Deleted: -
Deleted: ¶ ¶

Supplementary Table 4. Results from a one-way ANOVA comparing linear regression models that used either [demographic variables + cortical lesion data] or [demographic + cortical lesion data + cerebellar GMV] to account for variation of treatment-related language gains (i.e., changes in the ability to name visually presented objects, i.e., *Treated40* scores). Inclusion of cerebellar GMV significantly improved model fit.

Formatted: Font: Bold

Deleted:

Deleted: in

Model Summary - Treated40Gains

Model	R	R^2	Adjusted R^2	RMSE
H_0	0.453	0.205	0.149	4.049
H_1	0.786	0.618	0.309	3.648

Note. Null model includes AGE, daysPostStroke, LesionSizeVoxels, RACE, SEX

ANOVA ▼

Model		Sum of Squares	df	Mean Square	F	p
H_0	Regression	300.667	5	60.133	3.667	0.005
	Residual	1164.216	71	16.397		
	Total	1464.883	76			
H_1	Regression	905.822	34	26.642	2.001	0.017
	Residual	559.061	42	13.311		
	Total	1464.883	76			

Note. Null model includes AGE, daysPostStroke, LesionSizeVoxels, RACE, SEX

Coefficients

							Collinearity Statistics	
Model		Unstandardized	Standard Error	Standardized	t	p	Tolerance	VIF
H_0	(Intercept)	7.846	6.512		1.205	0.232		
	AGE	−0.094	0.040	−0.255	−2.356	0.021	0.956	1.046
	daysPostStroke	-2.469×10^{-4}	2.790×10^{-4}	−0.098	−0.885	0.379	0.906	1.104
	LesionSizeVoxels	-1.569×10^{-5}	5.681×10^{-6}	−0.307	−2.761	0.007	0.906	1.104
	RACE	1.439	1.242	0.124	1.159	0.250	0.984	1.016
	SEX	−2.178	0.949	−0.247	−2.295	0.025	0.966	1.036
H_1	(Intercept)	−24.925	12.186		−2.045	0.047		
	AGE	−0.106	0.057	−0.289	−1.857	0.070	0.376	2.660
	daysPostStroke	-9.666×10^{-6}	3.310×10^{-4}	−0.004	−0.029	0.977	0.522	1.914
	LesionSizeVoxels	-1.339×10^{-5}	8.012×10^{-6}	−0.262	−1.672	0.102	0.370	2.705
	RACE	6.036	1.803	0.518	3.348	0.002	0.379	2.639
	SEX	−1.045	1.248	−0.119	−0.837	0.407	0.453	2.208
	GMV_Left_I_IV	−29.901	66.553	−0.137	−0.449	0.656	0.097	10.270
	GMV_Right_I_IV	−18.078	70.845	−0.090	−0.255	0.800	0.073	13.737
	GMV_Left_V	−61.847	57.747	−0.320	−1.071	0.290	0.102	9.848
	GMV_Right_V	209.755	65.419	1.239	3.206	0.003	0.061	16.444
	GMV_Left_VI	1.729	33.167	0.020	0.052	0.959	0.064	15.736
	GMV_Vermis_VI	−36.564	80.108	−0.085	−0.456	0.650	0.262	3.811
	GMV_Right_VI	−72.853	41.314	−0.856	−1.763	0.085	0.039	25.922
	GMV_Left_CrusI	−29.544	33.214	−0.524	−0.889	0.379	0.026	38.258
	GMV_Vermis_CrusI	−1308.571	1934.201	−0.083	−0.677	0.502	0.598	1.673
	GMV_Right_CrusI	32.687	32.590	0.595	1.003	0.322	0.026	38.745
	GMV_Left_CrusII	−77.227	37.745	−1.011	−2.046	0.047	0.037	26.877
	GMV_Vermis_CrusII	89.843	262.474	0.062	0.342	0.734	0.273	3.663
	GMV_Right_CrusII	52.768	37.681	0.767	1.400	0.169	0.030	32.980
	GMV_Left_VIIb	−5.197	49.765	−0.044	−0.104	0.917	0.052	19.218
	GMV_Vermis_VIIb	999.164	603.632	0.301	1.655	0.105	0.274	3.644
	GMV_Right_VIIb	8.935	57.644	0.076	0.155	0.878	0.038	26.159
	GMV_Left_VIIIa	−38.151	61.619	−0.290	−0.619	0.539	0.042	24.066
	GMV_Vermis_VIIIa	319.179	185.837	0.461	1.718	0.093	0.126	7.920
	GMV_Right_VIIIa	19.932	70.608	0.154	0.282	0.779	0.030	32.892
	GMV_Left_VIIIb	−67.960	59.123	−0.408	−1.149	0.257	0.072	13.862
	GMV_Vermis_VIIIb	−488.766	310.392	−0.442	−1.575	0.123	0.115	8.685
	GMV_Right_VIIIb	62.646	60.515	0.373	1.035	0.306	0.070	14.302
	GMV_Left_IX	−13.874	71.906	−0.077	−0.193	0.848	0.057	17.688
	GMV_Vermis_IX	177.213	219.459	0.267	0.807	0.424	0.083	12.013
	GMV_Right_IX	104.288	76.436	0.617	1.364	0.180	0.044	22.539
	GMV_Left_X	170.568	210.857	0.170	0.809	0.423	0.205	4.882
	GMV_Vermis_X	−457.636	363.310	−0.262	−1.260	0.215	0.210	4.766
	GMV_Right_X	157.635	227.134	0.155	0.694	0.491	0.181	5.516
	GAsymmetryInd	154.205	133.012	0.981	1.159	0.253	0.013	78.811

SUPPLEMENTARY METHODS

Imaging

T1-weighted structural images collected from individuals with chronic stroke were enantiomorphically healed prior to Cat12 preprocessing. This technique, originally described by Nachev and colleagues (2008), involves replacing damaged areas in the lesioned hemisphere (LH in this case) with data from the healthy hemisphere (RH in this case). After healing, the image can be input into standard normalization procedures, which warp the individual's brain to any standard 3D brain template (i.e., MNI, SPM, FSL). Without the healing process, the normalization procedure typically fails catastrophically (especially for larger regions which deviate from probabilistic maps of WM and GM) and in unpredictable ways (Nachev et al., 2008). This approach has been used in multiple studies in stroke, including studies from our lab (Basilakos et al., 2019; Rorden et al., 2009; Suarez et al., 2020; Wilmskoetter et al., 2021; Yourganov et al., 2015, 2018), and is embedded in our stroke-specific image processing pipeline, *nii_preprocess* (Rorden et al., 2020).

Additional Analyses

We conducted a series of exploratory Pearson's correlations to examine the relationship between demographic data and our primary dependent variables of interest, cerebellar volume, and asymmetry as illustrated in Supplementary Tables 1A and 1B. In all analyses subsequently reported we controlled for age, gender, and race.

Language Measures

Western Aphasia Battery (WAB)

Deleted: ¶
¶
¶
¶
¶
¶

Deleted: :

Deleted: ¶

Deleted: ¶

Deleted: ,

Deleted: ¶

Deleted: ¶

Deleted: ¶

For all C-STAR treatment studies, aphasia severity was computed by trained speech-language pathologists based on administration of the Western Aphasia Battery (WAB) or Western Aphasia Battery—Revised (WAB-R; Kertesz, 2022). Total WAB scores, as well as subscores for spontaneous speech, object naming, speech repetition, and speech comprehension, were calculated according to the manual. WAB scores were typically measured at intake (participant recruitment) in order to ensure recruits were eligible to participate in available studies that accepted only participants with WAB total scores indicating presence of aphasia.

Object Naming Task (Treated40)

The *Treated40* variable represents treatment related gains experienced by participants in various C-STAR intervention studies (*N* = 77). Participants included in this group completed sparse fMRI scanning sessions at multiple time points at the McCausland Center for Brain Imaging (MCBI) located at Palmetto Richland Heart Hospital, Columbia, SC. During these 10 min, 30 s sparse fMRI scans, individuals with chronic stroke were presented with 40 pictures of concrete objects and 40 pictures of abstract art. Pictures were viewed through a mirror placed directly above participants' eyes. Participants were instructed to name objects they recognized and remain silent when abstract pictures were presented. Speaking occurred during the off period of the scans (silent periods) and fMRI images were collected during quiet periods. Audio recordings made during the silent portion of the sparse fMRI scans, acquired using a Serene Sound MRI Compatible Audio System, were manually scored by trained speech-language pathologists offline. *Treated40* accuracy gains were calculated as

[# pictures correctly identified after treatment] – [# of pictures correctly identified before

Deleted: ¶
Deleted: -
Deleted: they
Deleted: which only
Deleted: ¶
Deleted: ¶
Formatted: Font: Italic
Deleted: -
Deleted: minute
Deleted: thirty-second
Deleted: the
Deleted: :
Deleted: ¶
Deleted: -

treatment]

with higher numbers indicating greater pre–post improvement. Additional details regarding the *Treated40* task, its administration, its and properties can be found in Fridriksson et al. (2009).

Deleted: ¶

Deleted: -

Deleted: here

Deleted: (

Deleted: ,
